# Association between stride length variability and osteosarcopenia in community-dwelling older women: a cross-sectional study

**DOI:** 10.1186/s12877-026-07995-6

**Published:** 2026-08-01

**Authors:** Youngyun Jin, Kwonseok Han, Donghyun Kim

**Affiliations:** 1 Department of Sport Science, Sungkyunkwan University, 2066, Seobu-ro, Jangan- gu, Suwon-si, 16419 Republic of Korea; 2 Department of Sport and Health Science, Hanbat National University, 125, Dongseo-daero, Yuseong-gu, Daejeon, 34158 Republic of Korea; 3 Department of Big Data and Healthcare Convergence, Hanbat National University, 125, Dongseo-daero, Yuseong-gu, Daejeon, 34158 Republic of Korea

**Keywords:** Osteosarcopenia, Gait variability, Stride length, Body mass index, Older women

## Abstract

**Background:**

Osteosarcopenia, the coexistence of sarcopenia and low bone mass, raises the risk of falls, fractures, and functional decline, yet its diagnosis depends on dual-energy X-ray absorptiometry (DXA) and muscle function testing, which are difficult to apply routinely in the community. Whether gait variability, a marker of neuromuscular control, is associated with osteosarcopenia and adds information beyond mean gait measures is unknown in Korean community-dwelling older women. We examined this association and the discriminative ability of gait variability for osteosarcopenia.

**Methods:**

In this cross-sectional study, 190 community-dwelling women aged 65 years or older were classified by DXA and the 2019 Asian Working Group for Sarcopenia criteria into normal (*n* = 32), osteoporosis (*n* = 118), and osteosarcopenia (*n* = 40) groups. Spatiotemporal gait parameters and their trial-to-trial variability, expressed as the coefficient of variation (CV), were measured during overground walking with the OptoGait system. Binary logistic regression and receiver operating characteristic (ROC) analyses were performed.

**Results:**

From the normal to the osteosarcopenia group, gait speed and stride length decreased while stride length CV increased. After adjustment for age and confounders, shorter stride length, higher stride length CV, and lower body mass index (BMI) were each independently associated with osteosarcopenia (all *p* ≤ 0.013). A combined model of these three variables discriminated osteosarcopenia with an area under the curve of 0.83 (sensitivity 75.0%, specificity 81.3%).

**Conclusions:**

In this cross-sectional sample, gait variability, combined with stride length and BMI, was associated with osteosarcopenia and may offer a practical, noninvasive screening adjunct to prioritize older women for confirmatory DXA assessment. Prospective validation is needed.

**Supplementary Information:**

The online version contains supplementary material available at https://doi.org/10.1186/s12877-026-07995-6.

## Background

The global acceleration of population aging has led to a substantial increase in the burden of age-related musculoskeletal disorders [1]. Osteosarcopenia, defined as the coexistence of sarcopenia and low bone mass (encompassing osteopenia and osteoporosis), is increasingly recognized as a distinct geriatric syndrome that poses a greater clinical threat than either condition alone [2]. A recent comprehensive meta-analysis reported that osteosarcopenia significantly increases the risks of fractures, falls, and mortality in older adults [3, 4].

The prevalence of osteosarcopenia in community-dwelling older adults varies across populations. In Korea, a prevalence of approximately 19.2% has been reported among community-dwelling adults aged ≥ 60 years, with a markedly higher prevalence in women [5]. This sex-based difference is largely attributable to the accelerated decline in bone density and muscle mass following menopause [6].

In addition to its association with increased fall and fracture risk, osteosarcopenia has been linked to reduced physical function, impaired activities of daily living, frailty, and lower quality of life [7, 8]. Therefore, early identification of osteosarcopenia in community settings is essential [9, 10]. However, the current diagnostic approach requires combined assessment of bone mineral density, muscle mass, muscle strength, and physical performance, which limits its routine application in primary care and community-based health programs [11]. In particular, dual-energy X-ray absorptiometry (DXA), although regarded as the reference standard for evaluating bone and muscle mass, is limited by accessibility and cost constraints. It does not capture qualitative muscle changes such as intramuscular fat infiltration [12, 13].

Therefore, the development of simple, non-invasive functional markers that can capture the combined musculoskeletal deterioration characteristic of osteosarcopenia remains an important clinical challenge.

Gait variability, defined as stride-to-stride fluctuations in spatiotemporal gait parameters, is recognized as a sensitive indicator of neuromuscular control deficits and an independent predictor of falls in older adults [14]. Increased gait variability has been observed in postmenopausal women with low bone mineral density [15] and in older adults with sarcopenia, who show altered lower-extremity neuromuscular activation during functional tasks [16], even in the absence of differences in gait speed. Furthermore, older adults with osteosarcopenia have been reported to exhibit poorer gait and balance performance than those with osteopenia or osteoporosis alone [17].

Taken together, these findings suggest that gait variability may reflect the combined musculoskeletal and neuromuscular deterioration of osteosarcopenia, even when mean gait measures remain within normal ranges.

However, prior gait research in this field has focused largely on mean spatiotemporal parameters, and whether gait variability adds discriminative information beyond mean values, particularly in Korean community-dwelling older women, who have lower muscle mass and physiological reserve than Western populations, has rarely been tested. We therefore examined the association of spatiotemporal gait parameters and their variability with osteosarcopenia, and evaluated the discriminative ability of the identified variables, individually and in a combined model with body mass index, using ROC analysis.

## Methods

### Study design and participants

This cross-sectional study initially recruited 300 community-dwelling older adults aged ≥ 65 years from welfare centers in Suwon, Republic of Korea, between September 2022 and October 2024. During initial screening, 16 individuals were excluded because of severe cognitive impairment or dementia (*n* = 8), musculoskeletal disorders affecting gait (*n* = 6), or a history of cardiovascular conditions (*n* = 2). The remaining 284 participants underwent the main evaluations, including body composition analysis using DXA, gait assessment, and physical fitness testing. Following data collection, an additional 42 participants were excluded because of incomplete DXA scans (*n* = 28), missing gait or fitness data (*n* = 11), or voluntary withdrawal (*n* = 3). Consequently, 242 participants with complete data were included in the study.

Written informed consent was obtained from all individuals prior to participation. The study was conducted in accordance with the Declaration of Helsinki and was approved by the Institutional Review Board of Sungkyunkwan University (SKKU 2022-08-039).

### Measurements

#### Anthropometrics and body composition

Height, weight, and whole-body composition were measured using DXA (QDR 4500 A; Hologic, Waltham, MA, USA). Body mass index (BMI) was calculated as weight (kg) divided by height squared (m²). Waist circumference was measured at the midpoint between the lower border of the rib cage and iliac crest. Appendicular skeletal muscle mass (ASM) was derived from DXA as the sum of lean soft tissue masses in the four limbs. Appendicular skeletal muscle mass index (ASMI) was calculated as ASM (kg) divided by height squared (m²). Bone mineral density of the femur total was obtained from the same DXA scan, and the corresponding T-score was used to classify bone status.

#### Spatiotemporal gait parameters

Spatiotemporal gait parameters were assessed using an OptoGait optical measurement system (Microgate Corporation, Bolzano, Italy). The system consists of parallel transmitting and receiving bars placed 1 m apart, which detect interruptions in the optical signal when the foot contacts the recording area, and automatically calculate gait parameters. The validity of this system for gait assessment in older adults has been previously established [18].

Participants walked along a 10 m walkway at a self-selected comfortable pace. The OptoGait system was positioned over the middle 5 m to capture steady-state gait, while the initial and final 2.5 m served as acceleration and deceleration zones. Each participant completed three walking trials. For each trial the system computed the mean of every spatiotemporal parameter, and the number of strides captured within the 5-m steady-state zone was recorded (mean 4.4 ± 0.6 strides per trial; 13.0 ± 1.7 across the three trials). Recorded parameters included gait speed (m/s), stride length (cm), cadence (steps/min), and double support time (% of gait cycle). For each parameter, the mean across the three trials represented typical performance, and gait variability was quantified as the trial-to-trial coefficient of variation (CV, %), defined as the standard deviation across the three trial values divided by their mean, multiplied by 100. Stride counts did not differ across groups and were unrelated to stride length variability (Supplementary Table S2).

#### Physical fitness

Physical fitness was evaluated using the Senior Fitness Test [19]. For the Timed Up and Go (TUG) test, participants rose from a seated position, walked 2.44 m (8 ft), turned, walked back, and sat down as quickly as possible. Two trials were performed, and the best time (s) was recorded. The 30-s chair stand and 30-s arm curl tests were also administered. A 5-lb (2.27 kg) dumbbell was used for the arm curl test in women.

#### Osteoporosis and sarcopenia

Sarcopenia was diagnosed according to the 2019 Asian Working Group for Sarcopenia (AWGS) criteria [20], defined as low muscle mass accompanied by reduced muscle strength and/or poor physical performance. Low muscle mass was determined using an ASMI cut-off of < 5.4 kg/m² for women.

Handgrip strength was measured using a digital hand dynamometer (TKK 5401; Takei Scientific Instruments Co., Ltd.). Two trials were performed on each hand, and the highest value (kg) was used, with a cut-off value of < 18 kg for women.

Physical performance was assessed using the Short Physical Performance Battery, which includes a standing balance test, 4-m gait speed test, and five-repetition sit-to-stand test [21]. Total scores range 0–12, with ≤ 9 indicating poor physical performance. The 4-m gait speed within the SPPB was obtained from a separate short-distance test and used solely for sarcopenia classification, distinct from the OptoGait gait speed analysed as a study parameter.

Low bone mass was defined as a femur total T-score ≤ − 1.0, encompassing both osteopenia and osteoporosis according to the World Health Organization classification [22]. This threshold has been used in previous studies on osteosarcopenia [7, 23], which is defined as the coexistence of sarcopenia and low bone mass. For brevity, women with low bone mass but without sarcopenia are hereafter referred to as the osteoporosis group, although this group also included women with osteopenia. Applying these criteria to the 242 participants, only 2 of the 47 men (4.3%) met the definition of osteosarcopenia. Given this very low prevalence, which was insufficient for stable statistical comparison, men were excluded from further analysis. This decision was made a priori based on prevalence grounds and was independent of any study outcomes. Among the 195 women, five with sarcopenia but without low bone mass were excluded because the subgroup (*n* = 5) was too small for stable comparison. The final sample comprised 190 women, classified into three groups: normal (*n* = 32), osteoporosis (*n* = 118), and osteosarcopenia (*n* = 40).

#### Covariates

Systolic and diastolic blood pressures were measured using an automated sphygmomanometer (FG-500R; Jawon Medical, Republic of Korea) after at least 5 min of seated rest. Physical activity was assessed using the International Physical Activity Questionnaire-Short Form [24, 25], with total scores expressed as metabolic equivalent of task-minutes per week. Data regarding age, years of education, number of medications, smoking status (ever/never), and alcohol consumption were collected through face-to-face interviews with trained researchers. Fall-related self-efficacy was assessed using the Korean version of the Falls Efficacy Scale [26].

### Statistical analysis

Continuous variables are presented as mean ± standard deviation, and categorical variables as frequency (%). Participant characteristics were compared across the three ordered groups using linear-by-linear (trend) tests for continuous variables and chi-square tests for categorical variables; p-values for trend are reported in Table 1.

Binary logistic regression was used to examine associations between gait parameters and osteosarcopenia. In the initial stage, each predictor was analyzed individually, with adjustment for age (Model 1). Subsequently, variables with *p* < 0.05 were integrated into a multivariate model, and backward elimination was implemented using a removal criterion of *p* > 0.10 (Model 2). Multicollinearity in Model 2 was assessed using variance inflation factors (VIFs). Model calibration was evaluated with the Hosmer–Lemeshow test, and model fit was summarized using the McFadden pseudo-R² and the Akaike information criterion. To account for multiple variable testing, the robustness of the main associations was further examined using a Bonferroni-corrected significance threshold.

Receiver operating characteristic curve analysis was conducted to evaluate the discriminative ability of the identified variables, individually and in combination, for osteosarcopenia. The optimal cut-off values were determined using the Youden index.

All analyses were conducted using SPSS version 23.0 (IBM Corp., Armonk, NY, USA), and statistical significance was set at *p* < 0.05.

## Results

### Participant characteristics

A total of 190 women were included in the analysis: 32 in the normal group, 118 in the osteoporosis group, and 40 in the osteosarcopenia group. Significant linear trends were observed for age, body composition, physical performance, and gait parameters across the groups (Table 1). Gait speed and stride length decreased progressively from the normal to osteosarcopenia groups (both *p* < 0.001), whereas double support time increased across the groups (*p* = 0.009). Gait variability measures also showed significant trends, with both gait speed CV and stride length CV increasing from the normal to osteosarcopenia groups (*p* = 0.012 and *p* = 0.004, respectively). Cadence and cadence CV did not differ significantly across groups (*p* ≥ 0.073). Stride counts per trial were similar across groups (Supplementary Table S2), indicating that the differences in gait variability were not attributable to differing stride numbers.

**Table 1:** Participant characteristics according to osteosarcopenia status (*N* = 190)

Variables	Normal(n=32)	Osteoporosis (n=118)	Osteosarcopenia (n=40)	*p*-value for trend
Age (years)	77.4±3.8	78.9±4.0	80.7±5.3	<0.001
Weight (kg)	65.1±6.7	56.3±8.1	53.8±5.8	<0.001
BMI (kg/m2)	28.0±3.0	24.7±3.3	23.4±2.6	<0.001
WC (cm)	102.9±11.5	97.1±14.6	95.2±12.9	0.023
ASMI (kg/m2)	6.0±0.6	5.7±0.5	5.1±0.5	<0.001
SBP (mmHg)	130.9±19.8	130.9±12.6	130.5±14.8	0.912
DBP (mmHg)	71.2±13.4	71.2±7.9	71.2±8.0	0.995
HGS (kg)	18.0±2.9	16.8±4.4	15.0±3.7	0.002
Arm curl (reps/30 s)	18.0±2.8	17.2±4.5	14.4±3.1	<0.001
Chair stand (reps/30 s)	14.2±2.9	13.9±3.8	10.3±2.1	<0.001
TUG (s)	7.2±1.1	7.4±1.6	8.0±2.6	0.043
Gait speed (m/s)	1.5±0.3	1.3±0.2	1.2±0.2	<0.001
Gait speed CV (%)	3.9±2.4	4.3±2.8	5.5±3.0	0.012
Stride length (cm)	113.0±8.5	110.8±10.3	101.6±9.9	<0.001
Stride length CV (%)	2.5±1.6	3.3±2.4	4.0±2.1	0.004
Cadence (steps/min)	121.4±8.6	122.0±10.5	117.4±11.5	0.076
Cadence CV (%)	2.0±1.3	2.4±1.6	2.7±2.0	0.073
Double support time (%)	25.0±2.8	25.2±3.2	26.9±3.6	0.009
Double support time CV (%)	5.3±3.3	6.1±4.5	6.5±3.1	0.254
Femur total T-score	‒0.2±0.5	‒2.0±0.7	‒2.1±0.6	<0.001
SPPB score	10.0±1.4	9.8±1.4	8.1±1.0	<0.001
TPA (MET-min/week)	990.4±770.6	860.6±852.7	550.0±608.3	0.017
FES score	93.9±8.7	88.6±12.9	81.5±10.6	<0.001
Number of medications	1.4±0.6	1.4±0.9	1.3±1.0	0.502
Alcohol consumption, n (%)	22 (68.8)	61 (51.7)	25 (62.5)	0.712
Ever smoker, n (%)	0 (0.0)	0 (0.0)	1 (2.5)	0.999

*BMI* body mass index, *CV *coefficient of variation, *WC *waist circumference, *ASMI* appendicular skeletal muscle mass index, *SBP* systolic blood pressure, *DBP *diastolic blood pressure, *HGS* handgrip strength, *TUG* Timed Up and Go, *SPPB *short physical performance battery, *TPA* total physical activity, *MET* metabolic equivalent of task, *FES* fall efficacy scale

### Factors associated with osteosarcopenia

Table 2 presents the results of logistic regression analysis for the factors associated with osteosarcopenia.

In the age-adjusted univariate analysis (Model 1), stride length (odds ratio [OR] = 0.919, *p* < 0.001), stride length CV (OR = 1.163, *p* = 0.049), gait speed CV (OR = 1.160, *p* = 0.015), cadence (OR = 0.961, *p* = 0.020), double support time (OR = 1.139, *p* = 0.020), arm curl (OR = 0.846, *p* = 0.001), and BMI (OR = 0.829, *p* = 0.002) were significantly associated with osteosarcopenia.

Following backward elimination, three independent predictors remained in the multivariate model (Model 2). Shorter stride length (OR = 0.897, 95% confidence interval [CI] 0.859–0.937, *p* < 0.001), higher stride length CV (OR = 1.241, 95% CI 1.046–1.471, *p* = 0.013), and lower BMI (OR = 0.793, 95% CI 0.691–0.911, *p* = 0.001) were independently associated with the condition. Multicollinearity was negligible (all VIFs ≤ 1.02), and the model was well calibrated (Hosmer-Lemeshow χ^2^ = 11.73, df = 8, *p* = 0.164; McFadden Pseudo-R^2^ = 0.237; AIC = 157.2).

**Table 2:** Logistic regression analysis for factors associated with osteosarcopenia (N = 190)

Variables	Model 1		Model 2	
	OR (95% CI)	*p-*value	OR (95% CI)	*p-*value
Stride length	0.919 (0.881‒0.959)	<0.001	0.897 (0.859‒0.937)	<0.001
Stride length CV	1.163 (1.001‒1.351)	0.049	1.241 (1.046‒1.471)	0.013
Gait speed CV	1.160 (1.029‒1.307)	0.015	‒	‒
Cadence	0.961 (0.929‒0.994)	0.020	‒	‒
Double support time	1.139 (1.020‒1.270)	0.020	‒	‒
TUG	1.130 (0.939‒1.360)	0.195	‒	‒
Arm curl	0.846 (0.764‒0.936)	0.001	‒	‒
BMI	0.829 (0.734‒0.936)	0.002	0.793 (0.691‒0.911)	0.001
TPA	0.999 (0.999‒1.000)	0.074	‒	‒

Multivariate model: R2 = 0.237, AUC = 0.830, AIC = 157.2. Model 1: adjusted for age (each variable entered individually). Model 2: multivariate model derived by backward elimination (removal criterion: *p *> 0.10)

*OR* odds ratio, *CI* confidence interval, *CV* coefficient of variation, *TUG* Timed Up and Go, *BMI* body mass index, *TPA* total physical activity, *R2* coefficient of determination, *AUC* area under the receiver operating characteristic curve, *AIC* Akaike information criterion

Odds ratios are per one-unit increase: per 1cm (stride length), 1 percentage point (stride length and gait speed CV), 1 step/min (cadence), 1% of the gait cycle (double support time), and 1 kg/m2 (BMI)

### Discriminative ability

Table 3 presents the diagnostic performance of the individual variables and their combinations for discriminating osteosarcopenia.

Receiver operating characteristic curve analysis identified optimal cut-off values of stride length ≤ 108.5 cm (area under the curve [AUC] = 0.775, sensitivity 80.0%, specificity 65.3%), stride length CV ≥ 3.8% (AUC = 0.632, sensitivity 57.5%, specificity 70.7%), and BMI ≤ 25.6 kg/m² (AUC = 0.665, sensitivity 87.5%, specificity 46.7%).

**Fig. 1:**
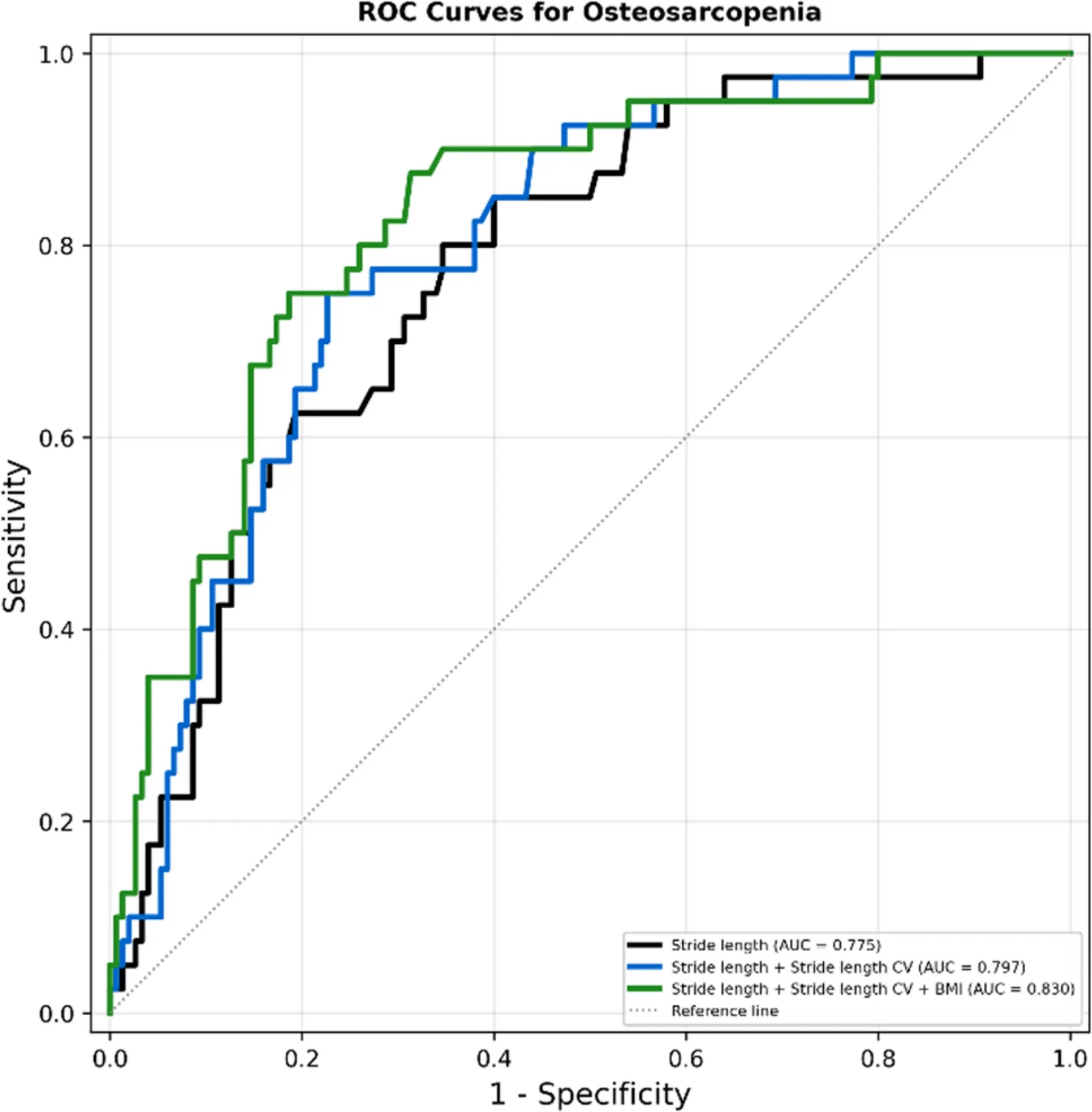
Receiver operating characteristic curves for discriminating osteosarcopenia. Black line represents stride length alone (AUC = 0.775, 95% CI 0.684‒0.865), blue line represents the combination of stride length and stride length CV (AUC = 0.797, 95% CI 0.710‒0.912), and green line represents the full model combining stride length, stride length CV, and BMI (AUC = 0.830, 95% CI 0.748‒0.921). AUC, area under the receiver operating characteristic curve; CI, confidence interval; CV, coefficient of variation; BMI, body mass index

When variables were combined sequentially, discrimination improved progressively. Stride length alone yielded an AUC of 0.775, with 80.0% sensitivity and 65.3% specificity. Adding stride length CV increased the AUC to 0.797 (sensitivity 75.0%, specificity 77.3%). The combined three-variable model (stride length, stride length CV, and BMI) provided the best discrimination (AUC = 0.830; sensitivity 75.0%, specificity 81.3%), exceeding any single variable (Fig. 1). Discriminative ability across the osteoporosis-versus-normal and osteosarcopenia-versus-osteoporosis is summarized in Supplementary Table S1.

**Table 3:** Receiver operating characteristic analysis for osteosarcopenia (N = 190)

	AUC (95% CI)	*p-value*	Cut-off	Sensitivity	Specificity
Individual variables					
Stride length	0.775 (0.684‒0.865)	<0.001	≤108.5	0.800	0.653
Stride length CV	0.632 (0.531‒0.734)	0.011	≥3.8	0.575	0.707
BMI	0.665 (0.565‒0.765)	0.001	≤25.6	0.875	0.467
Combined models					
Stride length	0.775 (0.684‒0.865)	<0.001	‒	0.800	0.653
Stride length + Stride length CV	0.797 (0.710‒0.912)	<0.001	‒	0.750	0.773
Stride length + Stride length CV + BMI	0.830 (0.748‒0.921)	<0.001	‒	0.750	0.813

*AUC* area under the receiver operating characteristic curve, *CI* confidence interval, *CV* coefficient of variation

## Discussion

The present study investigated the association between osteosarcopenia and spatiotemporal gait variability in community-dwelling older Korean women and evaluated the discriminative ability of gait variability parameters for identifying osteosarcopenia. We observed that gait speed and stride length decreased progressively from the normal to osteosarcopenia groups, whereas the gait speed CV and stride length CV increased progressively. After adjusting for age and other potential confounders, stride length, stride length CV, and BMI were independently associated with osteosarcopenia. The combined model incorporating these three variables yielded an AUC of 0.830, with 75.0% sensitivity and 81.3% specificity, suggesting acceptable discriminative ability.

A key finding was that stride length CV increased significantly across groups and remained independently associated with osteosarcopenia, even after adjustment for age and mean stride length. These findings suggest that stride-to-stride variability may reflect underlying neuromuscular control deficits not captured by conventional gait speed measures. This observation is consistent with findings from previous studies [16, 27–29]. Such variability likely reflects impairments in the bone-muscle-nerve axis [30]. Beyond mechanical coupling, biochemical crosstalk between bone and muscle mediated by myokines and osteokines is essential for maintaining neuromuscular junction stability [31]. In older Asian women, who typically have lower physiological reserves and muscle mass than their Western counterparts [32], disruption of this crosstalk may trigger neuromuscular junction fragmentation and functional denervation in the absence of overt mobility decline [31]. Overall, the increased gait variability observed in the osteosarcopenia group is unlikely to be solely a mechanical consequence of reduced muscle mass or bone density. It may instead reflect, at least in part, impaired neuronal regulation of the bone–muscle unit. Because neuromuscular function and myokine-osteokine signaling were not directly measured in this study, these interpretations should be regarded as hypotheses for future testing rather than established pathways. Future studies combining gait variability assessments with electrophysiological and biochemical markers are warranted.

Stride length emerged as the strongest single predictor of osteosarcopenia with an AUC of 0.775, consistent with previous reports. Jiang et al. [33] reported that older adults with sarcopenia exhibited significantly shorter stride lengths and slower gait speeds than healthy controls. Dostanpor et al. [34] demonstrated that walking speed and stride length were significantly associated with T-scores in postmenopausal women with low bone mineral density. Similarly, Sakazaki et al. [35] identified a positive correlation between gait speed and bone strength in community-dwelling postmenopausal Japanese women. These findings have been interpreted as reflecting a conservative gait strategy aimed at maintaining stability. When skeletal and muscular deficits coexist, their effects on gait function are likely additive. Szlejf et al. [36] demonstrated that, in middle-aged and older women, osteosarcopenic obesity is more strongly associated with impaired physical performance than either condition alone. Whereas these studies linked mean gait measures to sarcopenia or low bone mineral density separately, our findings extend them by showing that, where both deficits coexist, stride length variability is associated with osteosarcopenia independently of mean stride length.

In the present study, stride length showed a progressive decline across all three groups, supporting the notion that concurrent bone and muscle loss exerts a compounded effect on gait. In addition, double support time increased significantly across the groups alongside reduced stride length. This pattern may reflect a compensatory adaptation to maintain postural stability rather than a direct cause of falls [37].

Importantly, stride length CV remained independently associated with osteosarcopenia even after adjustment for stride length in the multivariate model, whereas gait speed CV was not retained despite being significant in the age-adjusted univariate analysis. Additionally, cadence and cadence CV did not differ significantly across groups. Because gait speed is a composite measure derived from the product of stride length and cadence, its CV likely reflects variability from multiple sources [38]. In contrast, stride length CV may more directly capture spatial control of stepping, which is linked to lower extremity muscle force generation and proprioceptive regulation [39]. This selective disruption of spatial parameters, with preserved temporal regularity, is consistent with the classical view that spatial and temporal gait parameters are governed by distinct control mechanisms [40]. It also suggests that the functional impact of osteosarcopenia is primarily driven by impaired lower extremity strength and balance rather than a generalized decline in central gait rhythm. Previous studies have reported that stride length CV can predict fall risk more effectively than conventional mean gait parameters and may serve as an indicator of functional decline [41–43]. Our findings corroborate and extend this evidence by demonstrating that stride length CV is also associated with osteosarcopenia and provides information that cannot be derived from mean stride length alone, as further supported by Shin et al. [44], who confirmed significant associations between gait parameters, muscle strength, and physical performance in older Korean adults. On its own, stride length variability demonstrated only modest discriminative ability (AUC = 0.63). Its primary value lies in the independent information it provides when combined with other variables, rather than as a stand-alone screening tool.

We also found that a lower BMI is independently associated with osteosarcopenia, which aligns with prior research identifying low body weight as a risk factor for both osteoporosis and sarcopenia. In a systematic review and meta-analysis, Du et al. [45] reported that BMI is significantly lower in older adults with osteosarcopenia than in those without. Lee et al. [46] similarly observed that lower BMI and physical inactivity are associated with osteosarcopenia in Korean community-dwelling adults aged ≥ 65 years. Despite its well-documented limitations as an index of body composition, BMI may retain clinical utility as a simple anthropometric indicator when combined with gait-based functional markers. This inverse association should be interpreted with caution. In older women with osteosarcopenia, a lower BMI primarily reflects reduced lean mass and bone mass rather than a protective effect of adiposity. Moreover, because BMI cannot differentiate between fat mass and lean mass, the relationship is likely non-linear at higher BMI values. However, osteosarcopenia has also been associated with an increased frailty risk independent of BMI [47, 48], suggesting that body composition alone does not fully explain the functional consequences of this condition.

From a clinical perspective, the three-variable model proposed in this study offers several practical advantages. All components can be assessed using a portable optical gait analysis system and a standard scale that does not require DXA. The OptoGait system used in the present study has been validated for spatiotemporal gait assessment in older adults [18], and a 10-m walking protocol is feasible in most community settings. Because the model was developed and tested within the same sample, the AUC of 0.830 represents an internal estimate that may be overly optimistic. Importantly, discriminative ability is a property of the combined three-variable model rather than of any individual parameter. Therefore, external validation in an independent cohort is needed before this approach can be recommended for screening in community health settings. For example, the model could be applied as a brief instrumented walk combined with routine body mass measurement. The relatively high specificity of the combined model may be particularly useful for ruling out osteosarcopenia and prioritizing individuals for further diagnostic evaluation using DXA. However, a sensitivity of 75.0% indicates that approximately one in four cases may be missed. Therefore, this model should be considered a screening adjunct rather than a definitive diagnostic tool. Future work should test whether gait variability precedes osteosarcopenia in prospective cohorts, validate the combined model in independent samples including older men, and integrate neuromuscular and biochemical measures to probe the bone–muscle–nerve hypothesis.

The present study has several limitations. First, its cross-sectional design precludes causal inference; we cannot determine whether increased stride length variability contributes to osteosarcopenia or is a consequence of it. All associations should therefore be interpreted as correlational. Second, the sample was limited to community-dwelling older women from welfare centers in a single city, and men were excluded due to the very low prevalence of osteosarcopenia (4.3%). Generalizability to men, other settings, or clinical populations is therefore limited. Third, important confounders such as cognitive function, peripheral neuropathy, vestibular dysfunction, pain, and vitamin D status were not measured. Thus, the observed associations should be considered partially adjusted. Fourth, the number of strides per trial was modest (mean 4.4), and variability was calculated using trial-to-trial rather than continuous stride-to-stride measures. In addition, combining osteopenia and osteoporosis into one group may have reduced contrast with the osteosarcopenia group. Fifth, the small and unbalanced group sizes (especially the normal group, *n* = 32), exclusion of participants with incomplete DXA data, and multiple variable testing may have reduced statistical power and increased the risk of type I error. However, the main associations remained robust after Bonferroni correction. Finally, because the model was developed and tested in the same sample, its discriminative performance (AUC = 0.830) may be overly optimistic and requires external validation in an independent cohort.

## Conclusions

In this cross-sectional sample, stride length variability was independently associated with osteosarcopenia, and a combined model of stride length, stride length variability, and BMI showed acceptable discriminative ability. These results provide preliminary evidence that gait variability may serve as a complementary, noninvasive screening marker to prioritize community-dwelling older women for confirmatory DXA assessment. Prospective, externally validated studies are needed before any diagnostic or causal value can be established.

## Supplementary information

Supplementary Material 1.

## Data Availability

The data associated with this paper are available from the corresponding author upon reasonable request.

## Abbreviations

ASMI: Appendicular skeletal muscle mass index
ASM: Appendicular skeletal muscle mass
AUC: Area under the receiver operating characteristics
AWGS: Asian Working Group for Sarcopenia
BMI: Body mass index
CI: Confidence interval
CV: Coefficient of variation
OR: Odds ratio
SPPB: Short Physical Performance Battery
TUG: Timed Up and Go
