# Supplementary material for: Association between stride length variability and osteosarcopenia in community-dwelling older women: a cross-sectional study
Source: BMC Geriatr. 2026 Aug 1;26:1129. doi: 10.1186/s12877-026-07995-6 (PMC13543560; doi:10.1186/s12877-026-07995-6)
Supplement: Supplementary file 1 [file 12877_2026_7995_MOESM1_ESM.docx]

**Supplementary Table S1.** Discriminative ability of stride length, stride length CV, and BMI for identifying osteosarcopenia, osteoporosis, and osteosarcopenia versus osteoporosis

| **Variable** | **AUC (95% CI)** | **Sensitivity (%)** | **Specificity (%)** |
| --- | --- | --- | --- |
| **Osteosarcopenia vs non-osteosarcopenia** (n = 190) | | | |
| Stride length | 0.775 (0.694–0.848) | 80.0 | 65.3 |
| Stride length CV | 0.632 (0.533–0.727) | 57.5 | 70.7 |
| BMI | 0.665 (0.579–0.748) | 87.5 | 46.7 |
| Combined model | 0.830 (0.756–0.893) | 75.0 | 81.3 |
| **Osteoporosis vs normal** (n = 150) | | | |
| Stride length | 0.565 (0.461–0.666) | 36.4 | 84.4 |
| Stride length CV | 0.589 (0.475–0.696) | 68.6 | 50.0 |
| BMI | 0.777 (0.692–0.860) | 74.6 | 75.0 |
| Combined model | 0.796 (0.710–0.871) | 80.5 | 75.0 |
| **Osteosarcopenia vs osteoporosis** (n = 158) | | | |
| Stride length | 0.759 (0.676–0.840) | 80.0 | 61.9 |
| Stride length CV | 0.612 (0.505–0.715) | 57.5 | 68.6 |
| BMI | 0.607 (0.512–0.696) | 80.0 | 44.9 |
| Combined model | 0.801 (0.717–0.876) | 75.0 | 78.8 |

AUC, area under the receiver operating characteristic curve; CI, confidence interval; CV, coefficient of variation; BMI, body mass index. Combined models include stride length, stride length CV, and BMI. Optimal cutoff values were determined using the Youden index. 95% CI estimated by 2000-iteration bootstrap.

**Supplementary Table S2.** Number of strides captured per walking trial by group

| Group | n | Trial 1 | Trial 2 | Trial 3 | Mean per Trial |
| --- | --- | --- | --- | --- | --- |
| Normal  Osteoporosis  Osteosarcopenia  Total | 32  118  40  190 | 4.17 ± 0.44  4.33 ± 0.62  4.53 ± 0.83  4.35 ± 0.65 | 4.22 ± 0.41  4.33 ± 0.55  4.52 ± 0.73  4.35 ± 0.58 | 4.22 ± 0.46  4.36 ± 0.58  4.51 ± 0.71  4.37 ± 0.59 | 4.20 ± 0.43  4.35 ± 0.58  4.52 ± 0.74  4.36 ± 0.60 |
| Values are mean ± standard deviation. Across the three trials, participants completed 13.0 ± 1.7 (range 10-21). Stride counts did not differ across groups (one-way ANOVA, *p* = 0.083) and were uncorrelated with stride length variability (Pearson r = 0.009, *p* = 0.904), | | | | | |
